# Supplementary figures and images for: Overexpression of βTrCP1 elicits cell death in cisplatin-induced senescent cells
Source: Cell Death Dis. 2025 Mar 25;16(1):203. doi: 10.1038/s41419-025-07556-6 (PMC11937513; doi:10.1038/s41419-025-07556-6)

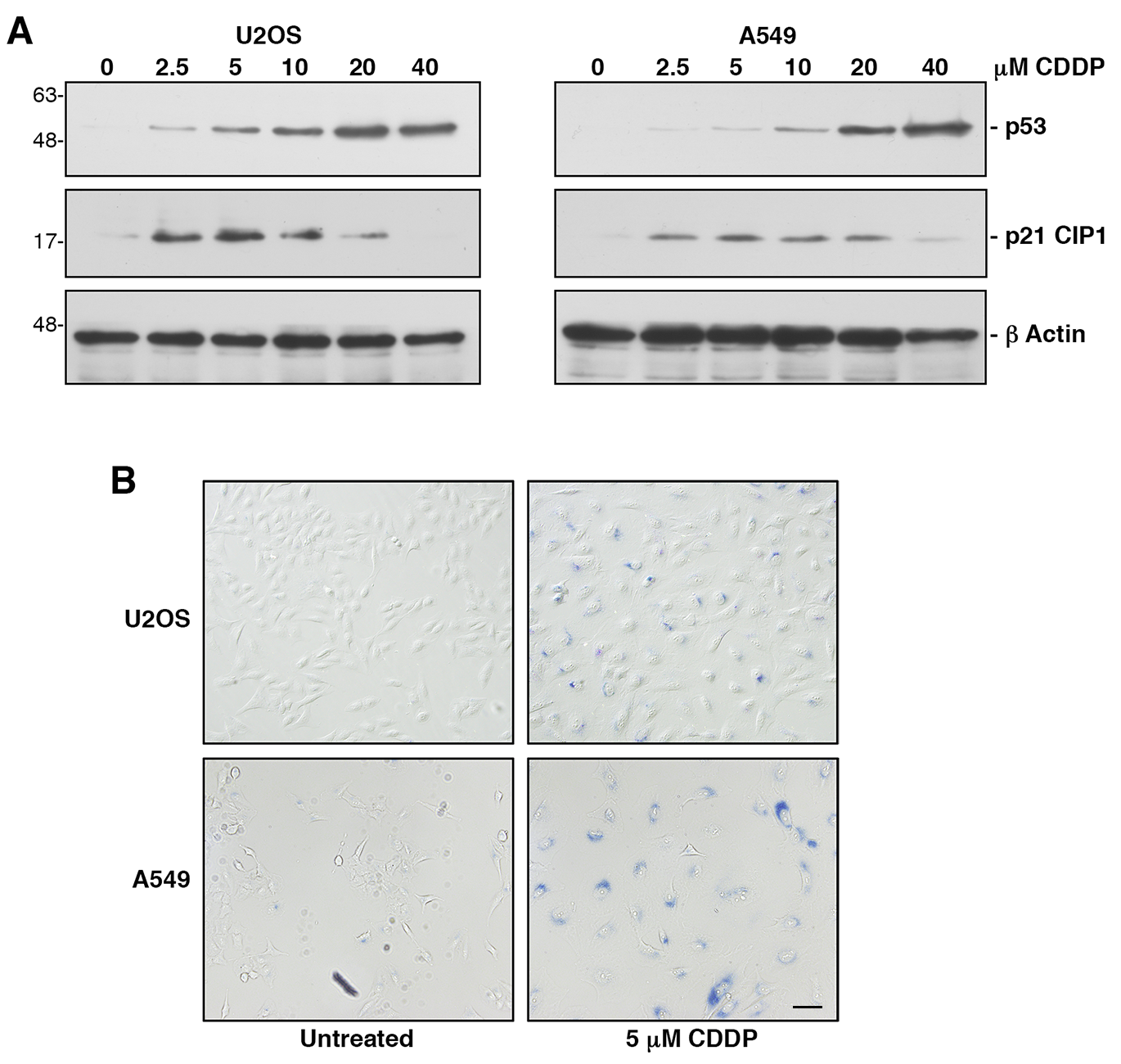

Supplement: Supplementary file 2 — Supplementary figure 1 [file 41419_2025_7556_MOESM2_ESM.tif]

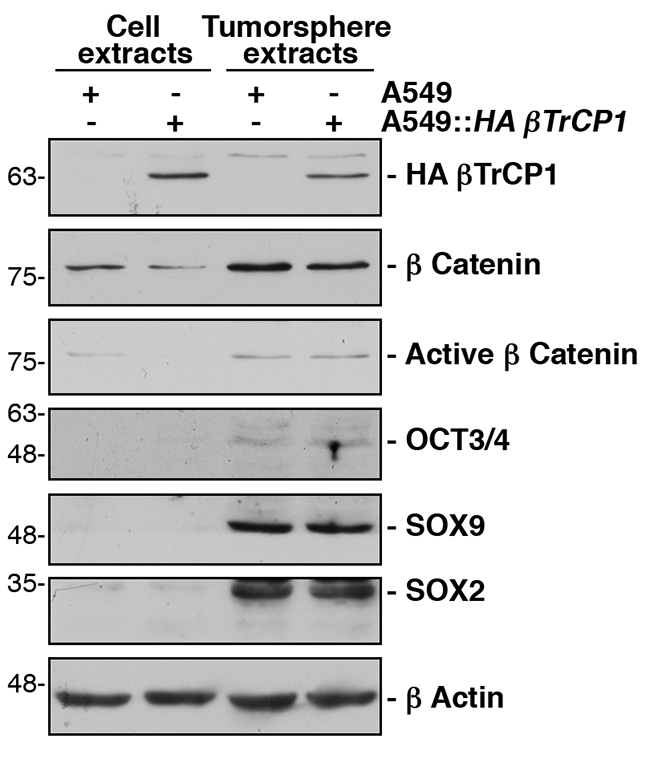

Supplement: Supplementary file 3 — Supplementary figure 2 [file 41419_2025_7556_MOESM3_ESM.tif]

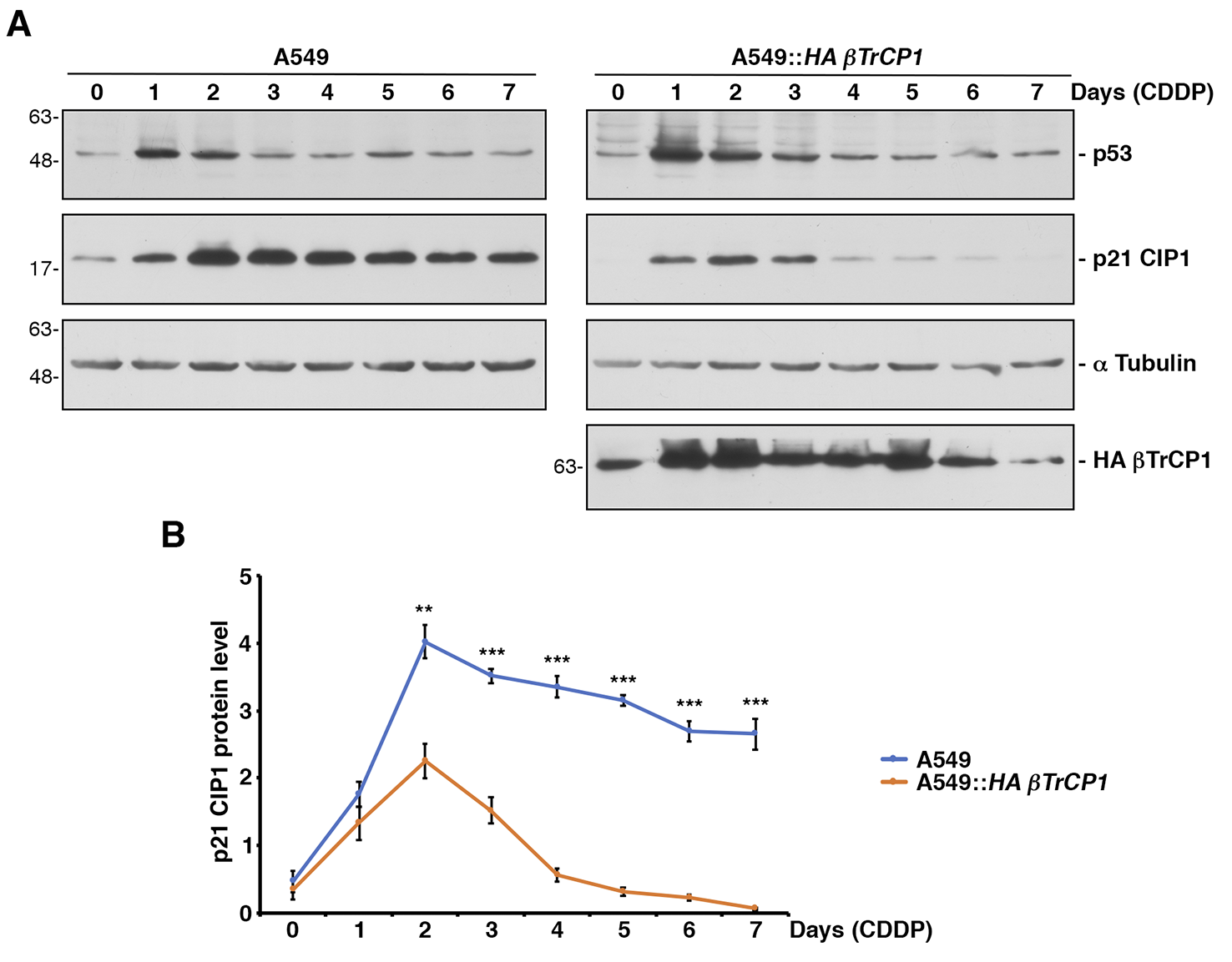

Supplement: Supplementary file 4 — Supplementary figure 3 [file 41419_2025_7556_MOESM4_ESM.tif]

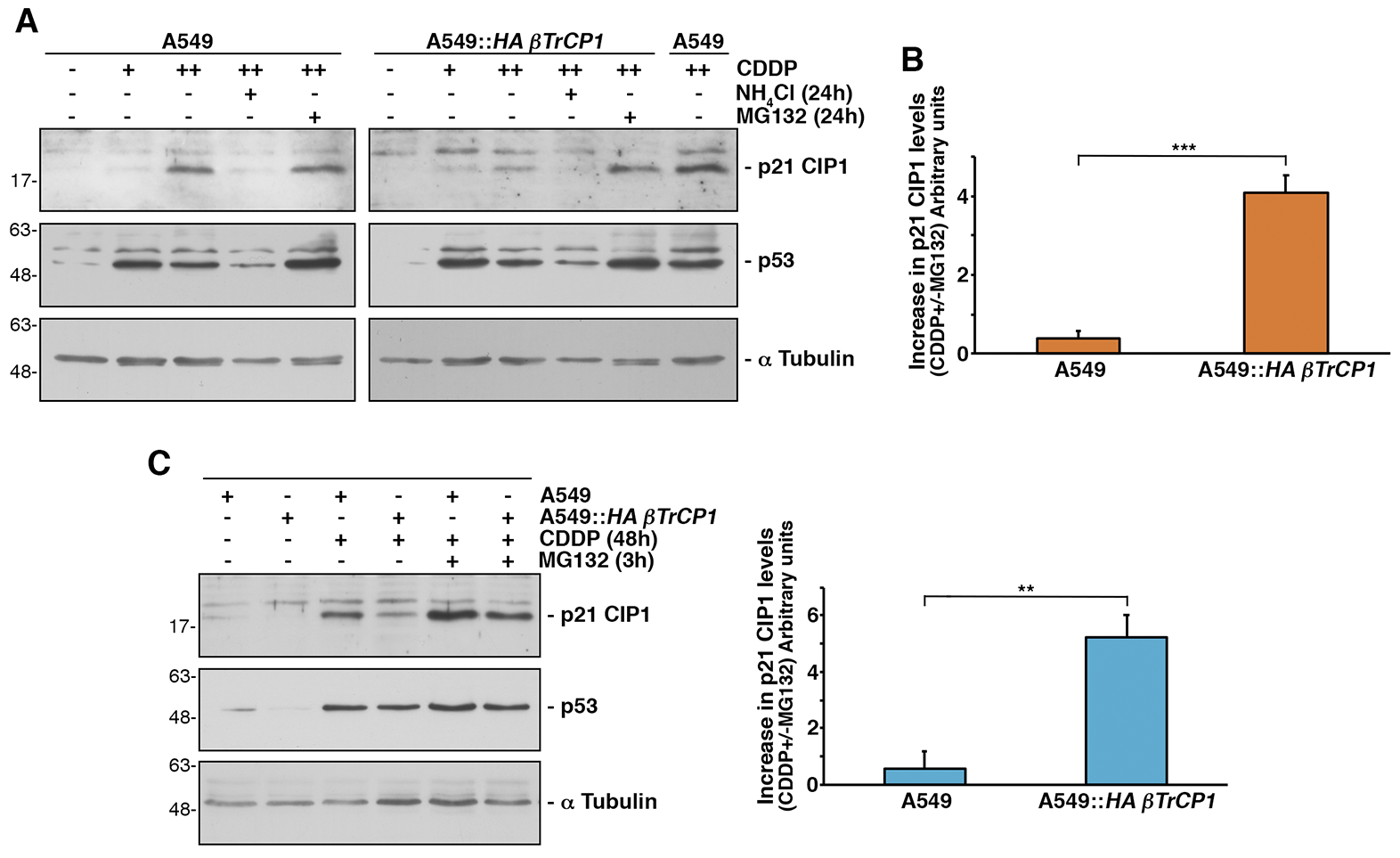

Supplement: Supplementary file 5 — Supplementary figure 4 [file 41419_2025_7556_MOESM5_ESM.tif]

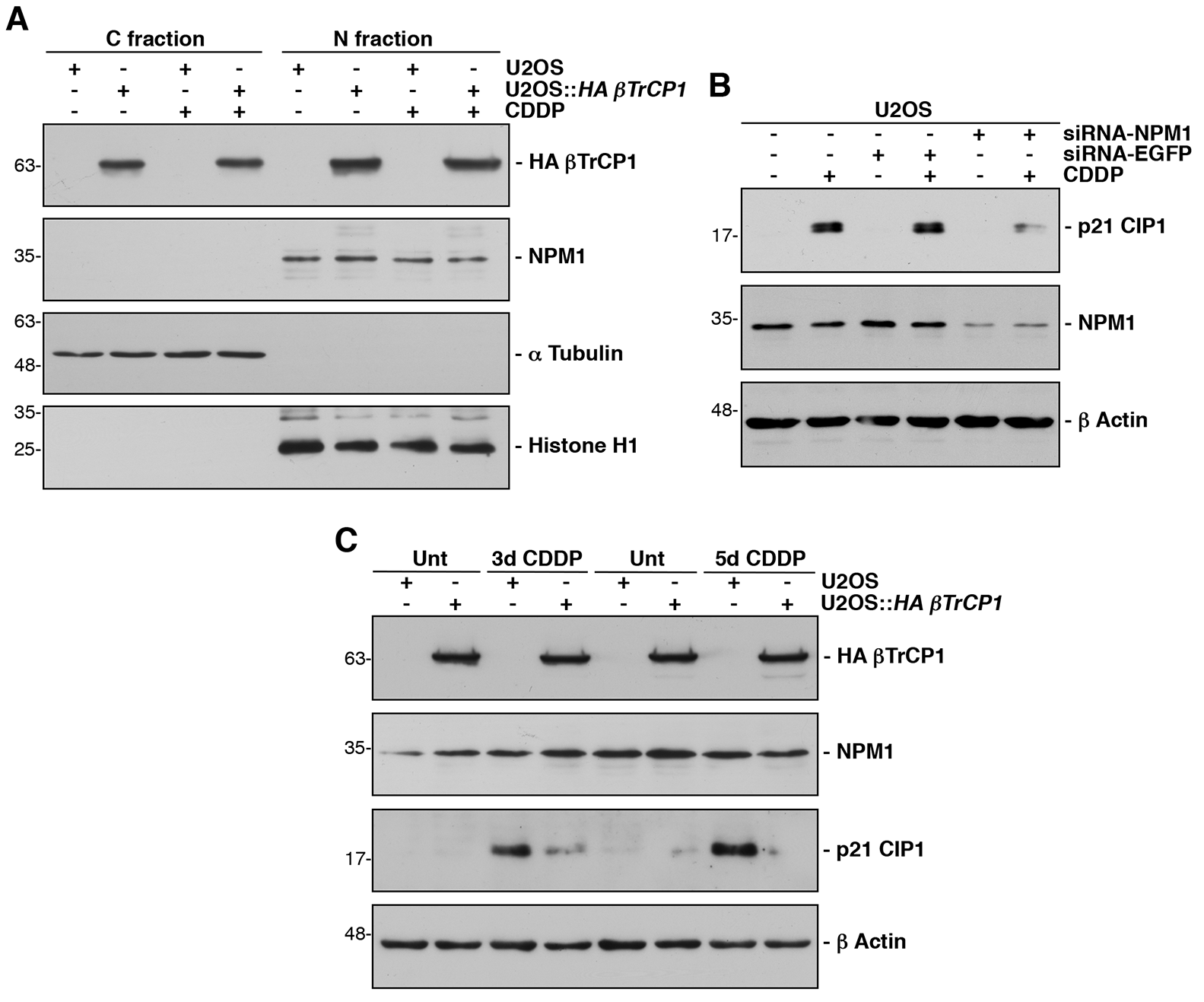

Supplement: Supplementary file 6 — Supplementary figure 5 [file 41419_2025_7556_MOESM6_ESM.tif]
