# Supplementary material for: Overexpression of βTrCP1 elicits cell death in cisplatin-induced senescent cells
Source: Cell Death Dis. 2025 Mar 25;16(1):203. doi: 10.1038/s41419-025-07556-6 (PMC11937513; doi:10.1038/s41419-025-07556-6)
Supplement: Supplementary file 8 — Original blots [file 41419_2025_7556_MOESM8_ESM.pdf]

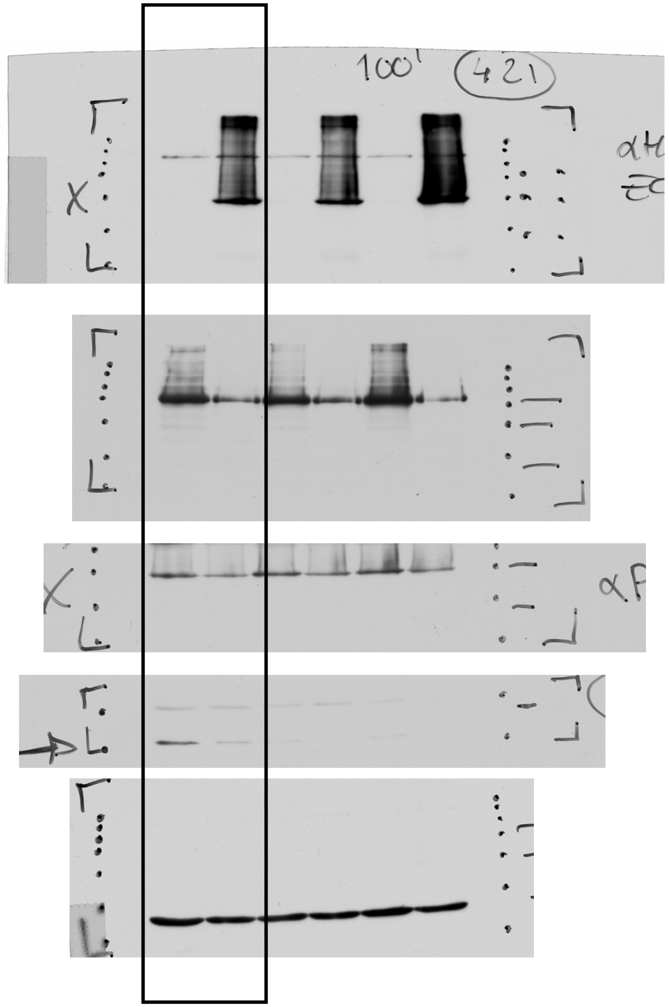

Figure 1B

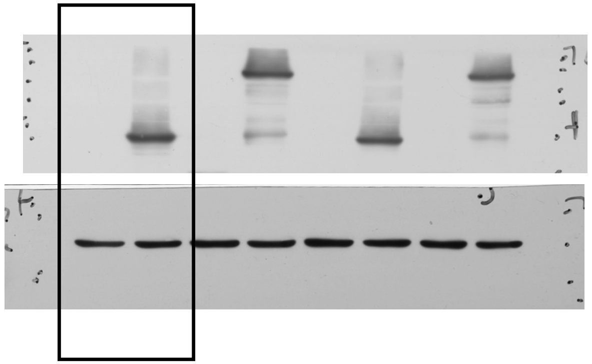

Figure 1D

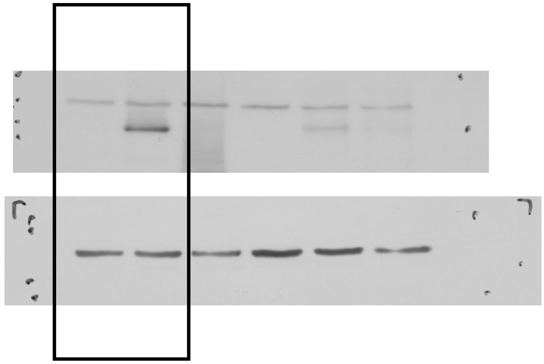

Figure 1E

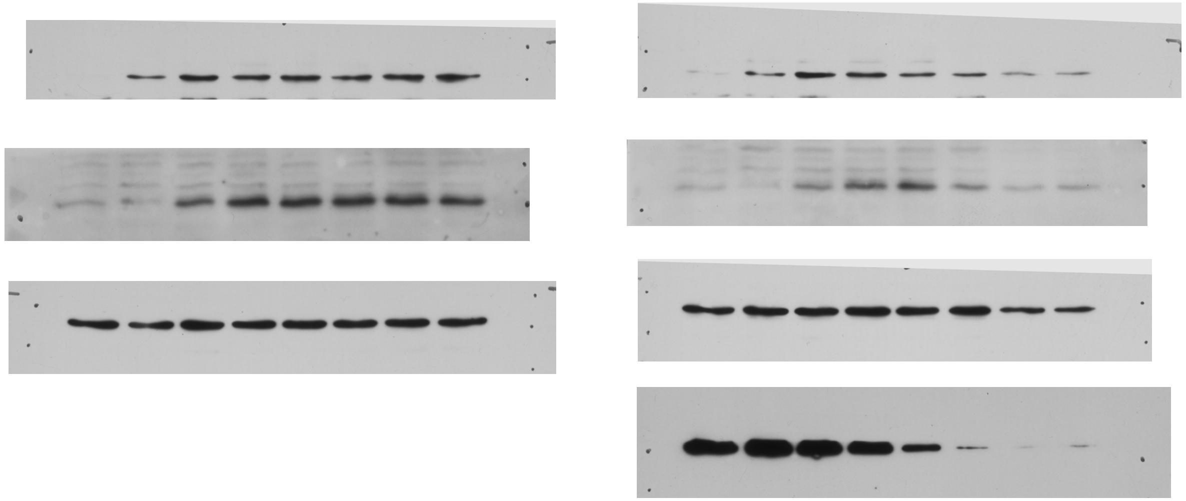

Figure 3A

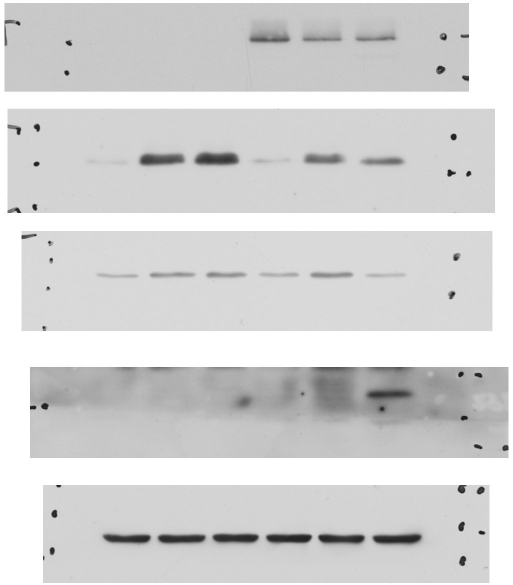

Figure 3E

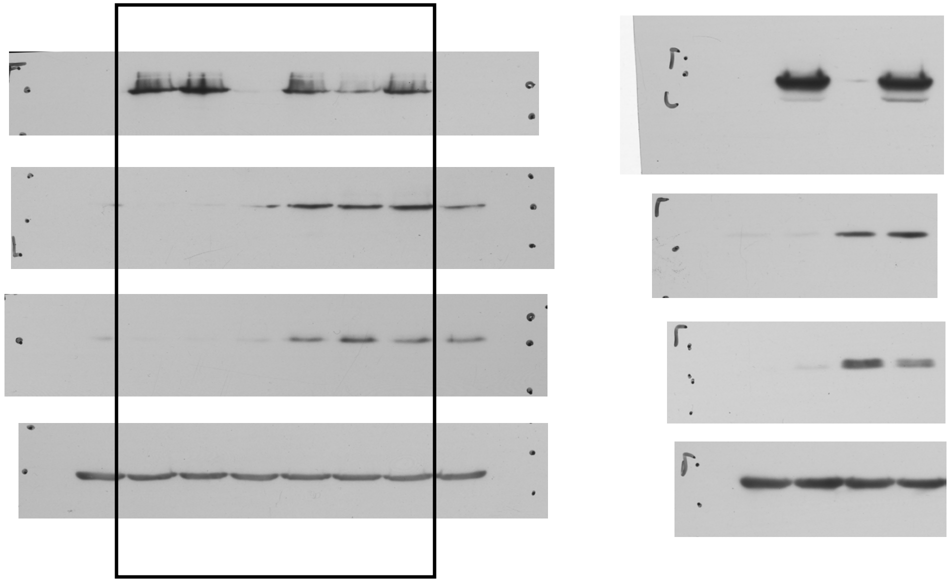

Figure 4A

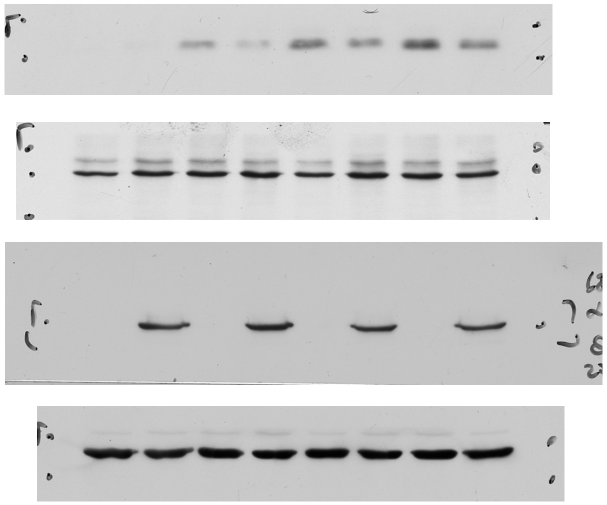

Figure 4C

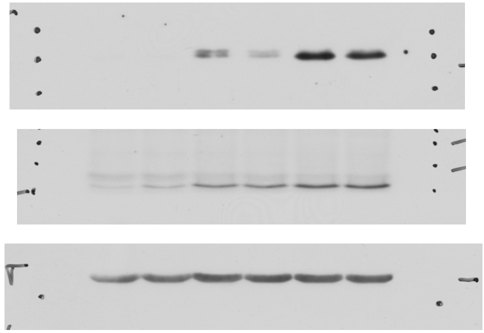

Figure 4D

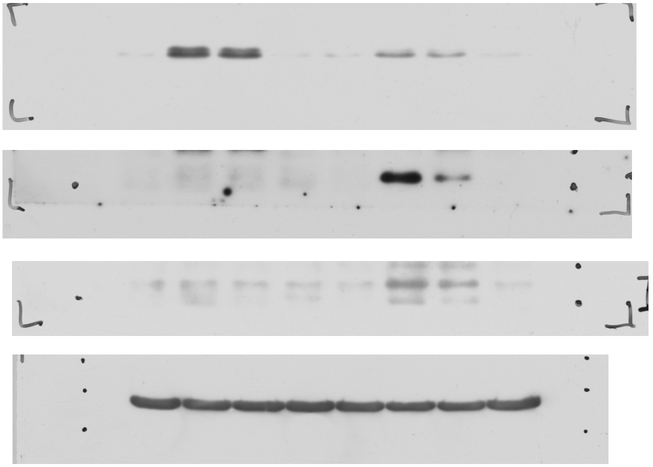

Figure 4E

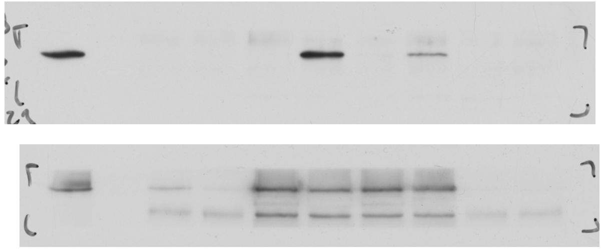

Figure 5A

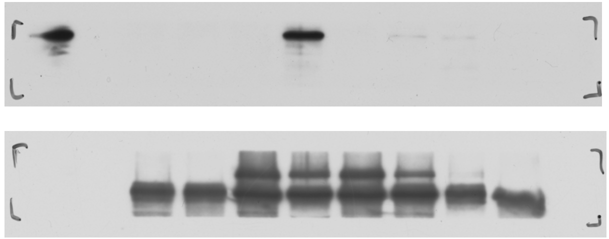

Figure 5B

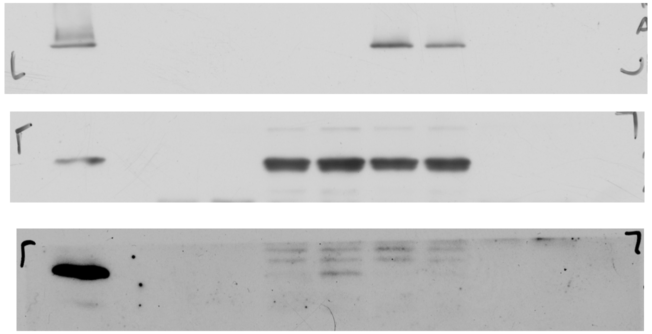

Figure 5C

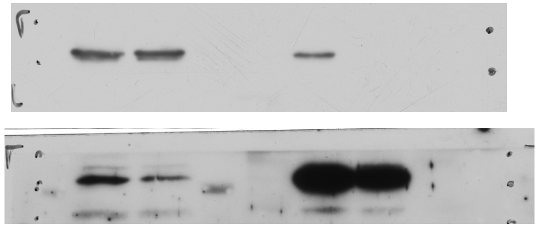

Figure 5D

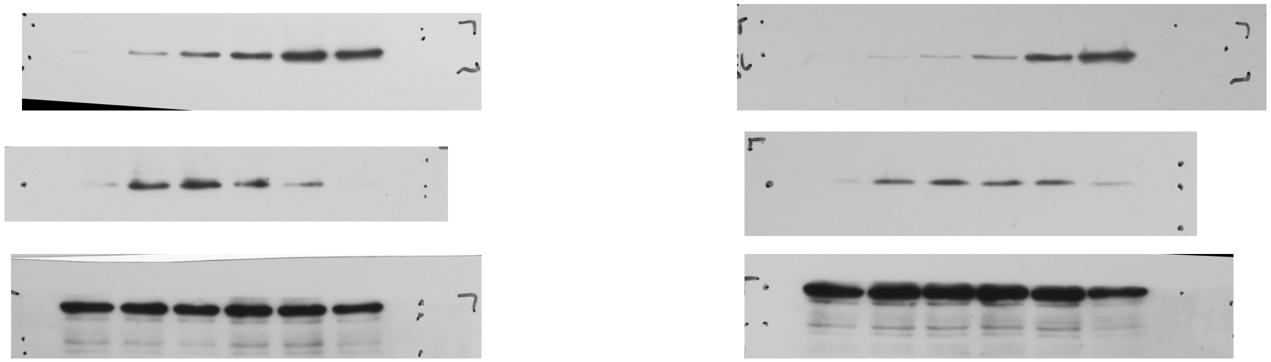

Suppl Figure 1A

Suppl Figure 2

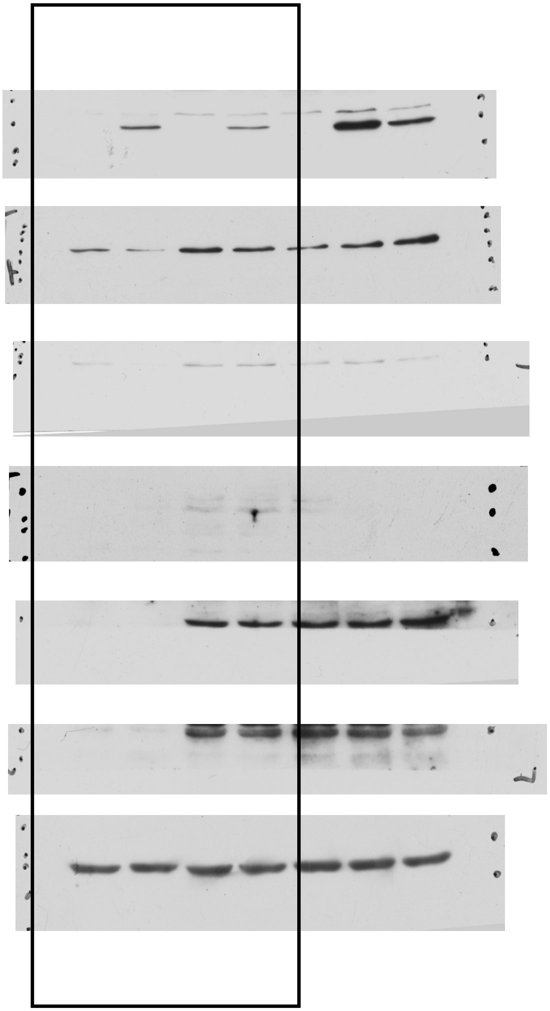

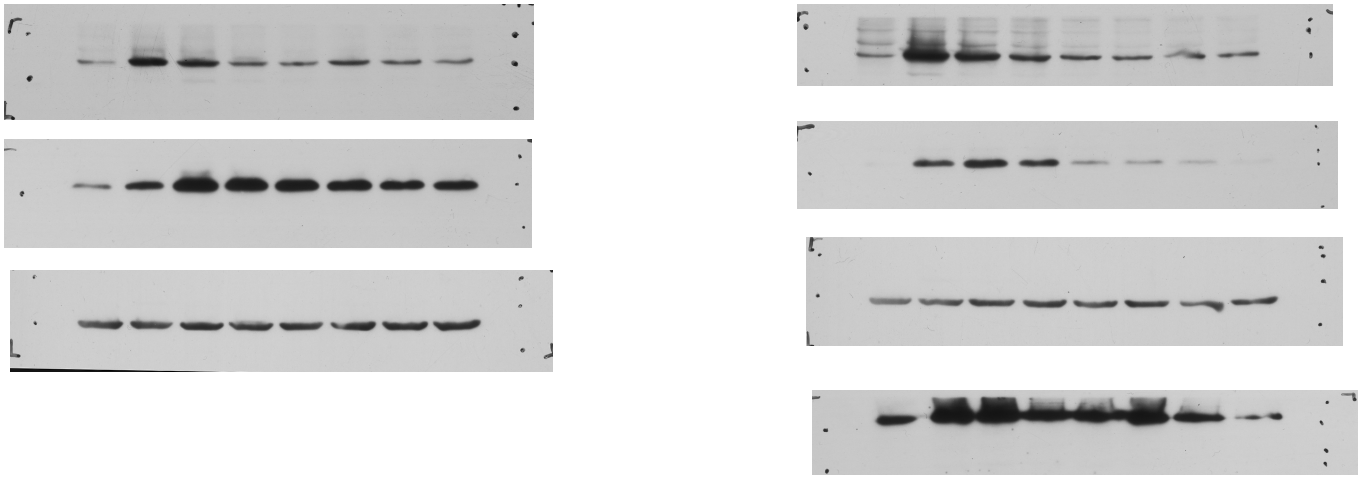

Suppl Figure 3A

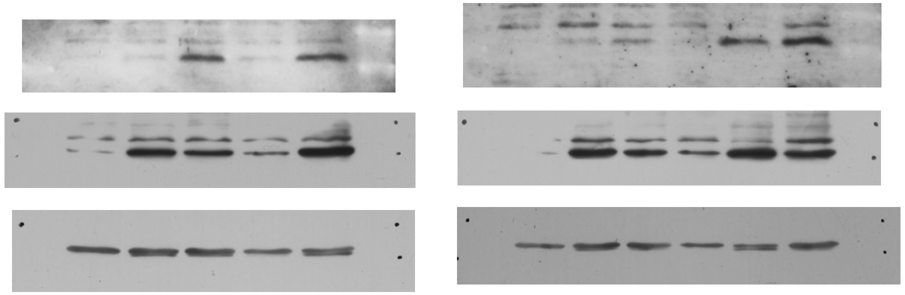

Suppl Figure 4A

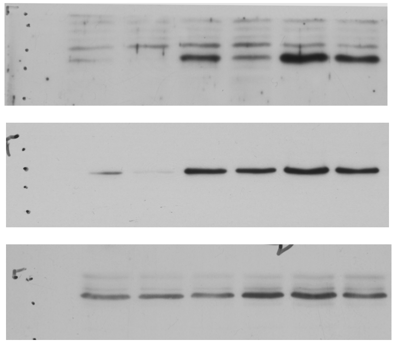

Suppl Figure 4C

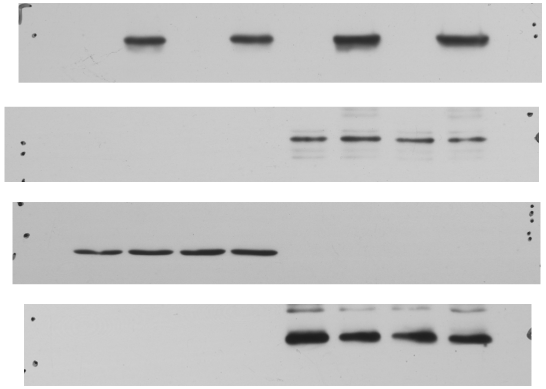

Suppl Figure 5A

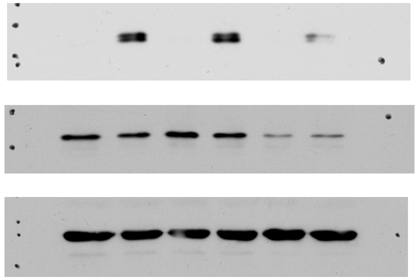

Suppl Figure 5B

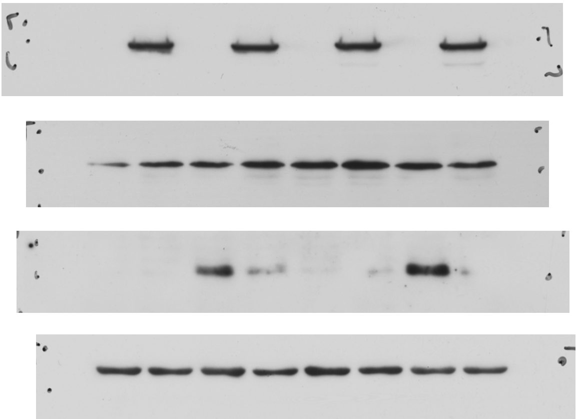

Suppl Figure 5C
